# Practical Remarks on That Cachectic State Which Is so Common a Precursor of Pulmonary Consumption

**Published:** 1826-11

**Authors:** Whitlock Nicholl


THE LONDON
### Medical and Physical Journal.

No 333, VOL. LVI.] NOVEMBER, 1826. [No 5, *New Series*.

For many fortunate discoveries in medicine, and or the detection of numerous errors, the world is indebted to the rapid circulation of Monthly Journals; and there never existed any work, to which the Faculty, in Europe and America, were under deeper obligations, than to the Medical and Physical Journal of London, now forming a long, but an invaluable, series.—RUSH.

### ORIGINAL PAPERS,

AND

### CASES OBTAINED FROM PUBLIC INSTITUTIONS AND OTHER AUTHENTIC SOURCES.

### PREVENTION OF PHTHISIS.

*Practical Remarks on that Cachectic State which is so common a Precursor of Pulmonary Consumption.* By WHITLOCK NICHOLL, M.D. M.R.I.A. F.L.S. &c. &c.

IF phthisis, or pulmonary consumption, be a disease so frequent "as to carry off prematurely (according to Dr. YOUNG's estimate) one-fourth part of the inhabitants of Europe, and so fatal that M. BAYLE will not allow it possible for any to recover who suffers from it in its genuine form,"* no apology need be offered for the communication of any suggestions, however trifling, which may, in the slightest degree, tend to prevent the occurrence of so frequent and so fatal a malady.

There is a certain assemblage of symptoms, which, if unopposed, or if ineffectually opposed, is seen, in a host of instances, to terminate in phthisis. A similar assemblage of symptoms, if attacked with energy in an early stage, may, in many cases, be entirely dispersed, no pulmonary affection supervening; and, although no positive evidence can be adduced to prove that phthisis would have been formed in these latter cases, if the symptoms had not yielded to the timely aid of medicine, yet we may fairly conclude that such would have been the termination of these cases also, if they had not been arrested in their career.

When the assemblage of symptoms to which I allude is followed by phthisis pulmonalis, it matters not whether we regard the pulmonary disease as the direct effect of that

* Dr. GOOD's Study of Medicine.

earlier indisposition which gave rise to those symptoms, or whether we say that the precursory indisposition merely roused into action (for this is the favourite phrase,) that pulmonary malady to which there was a strong predisposition. We are not contending about technical forms of speech, but we are discussing facts of vital importance. It is sufficient for us that we know that a certain disordered condition of the general system, as manifested by certain symptoms, is seen, in a great many instances, to lead to phthisis, and that a condition apparently similar in every respect may, in many cases, be removed by proper management.

In the remarks which I am about to offer, I do not pretend to advance any new doctrine, or to state any new fact in the history of maladies which terminate in phthisis; nor do I boast of the discovery of any new or specific remedy. But I wish to direct attention to that cachectic state of the system which is so common a precursor of phthisis, and to offer a summary of that combination of familiar means which I have found most efficacious in removing that precursory state.

The cachectic state which is so common a forerunner of phthisis, is marked by the following symptoms:—Languor, lassitude, loss of power and of energy, gradual emaciation, general pallor, coldness of the extremities; all these symptoms being present without any marked disorder in any particular organ. The appetite may be diminished. The general health seems, to use the popular phrase, to be breaking up. Yet, if you ask the patient how he is, he will scarcely admit that he is ill. To this state succeed—irregular attacks of slight febrile excitement; cough, which at first scarcely attracts notice, and of which the patient is hardly sensible; transient uneasiness about the chest, which is scarcely the subject of complaint; wakefulness, perhaps, and restlessness, for which no reason is assigned; with a tendency to increased perspiration. These are the symptoms which principally characterise that disordered state of the system, which, in so vast a number of instances, paves the way to confirmed phthisis. If such a cachectic condition be suffered to exist for any length of time, the symptoms will become aggravated, the signs of pulmonary distress will gradually develop themselves, and all those characters will be present which too plainly indicate the existence of incurable local disease.

The cachectic state which I am endeavouring to describe, is, in an early stage of its existence, curable in a large proportion of instances; whereas, the local disease to which this cachectic state leads, is incurable. Whenever, therefore, those symptoms which characterise this cachectic state present themselves, our utmost endeavours must, without loss of time, be exerted to remove this general indisposition of the system, before pulmonary disease be induced. It frequently happens, however, that the general cachexy manifests itself so gradually, and the formation of local disease is so silent and insidious, that the disorder of the system may have merged into pulmonic disease before the patient is aware of his perilous condition. But, if the patient fall under our observation even in the earlier stages of this insidious affection, a practised eye will, generally speaking, detect the mischief which is secretly unfolding itself. At all events, however strongly we may suspect that the lungs have become diseased, we must, with equal ardour, adopt and pursue those remedial means which prove salutary in the simple cachectic state, in the hope that our fears may prove unfounded, and that the disease may yet be within the control of medicine.

The cachectic state which leads to phthisis may be present at an early age; it is very commonly met with during adolescence, and in adults. It presents itself in persons of either sex, but it is especially seen in young females, and of these a large proportion will prove to be single women. Mental anxiety, disappointed hope, suppressed sorrows, and profound grief, are too often the slow causes of that general derangement of the health which so commonly, sooner or later, terminates in phthisis; and these mental emotions act with peculiar force on the delicate unresisting frame of a sensitive female. This cachectic state may also be a consequence of too rapid an elongation of the body at, or before, the season of puberty; it may result from undue confinement, and from over-exertion of the mental or bodily powers; it is often produced by too great a devotion to study: thus the present absurd fashion of forcing upon youthful females of all classes pseudo-accomplishments of every kind, tends, in no slight degree, to favour the production of this cachectic state. Unfortunately for the present and for future generations, the firm fibre of health and youth, the rosy tint which bespeaks energy and active life, and that spring of muscular activity which results from vigour of the general system, all these are denounced among females of fashionable life as the marks of coarseness and vulgarity; while nerveless inanition, pallor, and debility, are regarded as elegant marks of patrician birth and cultivated refinement. And, assuredly, few of our youthful females can run that career of studied dissipation and laborious pleasure which is chalked out in the paths of fashionable life for the delicate girl who has just escaped from the torturing hands of masters and finishers of every kind, without exhibiting, sooner or later, those symptoms which denote an enervated and exhausted constitution.

The cachectic state of which we are speaking may also result from defective or improper nutriment; from the respiration of a vitiated or over-heated atmosphere; from copious or repeated hemorrhage, whether spontaneous or artificial; from excessive excretions of various kinds, whether natural or morbid, and especially in females of a peculiar temperament, and in those of a delicate habit, from lactation too long continued. It may also be the consequence of high febrile action, and it may succeed to febrile diseases of the exanthe matous order. It may, in children or youthful persons of a peculiarly delicate and susceptible temperament, be induced by powerful doses of calomel frequently repeated; and it may, in persons of every age and temperament, be induced by long-continued courses of any preparation of mercury which produces in the system the specific effects of that mineral.

As the cachectic state which we are considering has a strong tendency to give rise to pulmonary disease, we must regard the lungs as the point towards which the several symptoms converge; so that, while the general indisposition of the whole system forms the leading object of our attention, our view must also be directed towards that point where local disease may be expected to occur. And this joint attention to the general derangement of the health, and to the common centre of attack, is the more necessary, because, although the prevention of local disease by the removal of the cachectic state forms the grand object of our endeavours, yet, as it is not possible to specify the exact point at which general indisposition passes into, and is blended with, local disease within the chest, that cachectic state may, in any given case, already have induced some incipient disease of the lungs, which, slight as it may be, may nevertheless have some share in heightening the signs of general disturbance, and may demand the more active employment of local means for the removal of it, and will assuredly, if it be not promptly removed, lay the firm foundation of permanent disease.

Our treatment, then, must embrace those general means which, by counteracting and removing the general cachectic condition, tend to prevent the formation or the development of local disease, and those local means which are calculated to suspend or remove that morbid condition of the lungs which may have been superadded to the constitutional disorder.

Suppose, then, that we are consulted by a patient who is in that cachectic state which I have endeavoured to describe,

—who labours under general asthenia,—whose spirits and whose energies are lowered or subdued,—whose size has been diminished by slow and imperceptible degrees,—who feels incapable of active exertion,—who is subject to coldness of the extremities, and to irregular attacks of slight febrile excitement and flushing of the countenance,—whose features are usually pallid, and have an expression of debility,—whose circulation is languid, the pulse, perhaps, being easily accelerated by bodily exertion or by mental emotion,—whose sleep is broken and unrefreshing,—who coughs from time to time, the cough being perhaps slight, or being at one time slight and at another time more violent,—who has some wandering or unfixed uneasiness about the chest, and whose respiration is perhaps hurried by walking or by any exertion of the body, or is at times slightly oppressed. Such a patient is to be regarded as journeying onward along the path which leads to phthisis; and, unless we instantly exert all those means which are calculated to arrest his progress, he may, in a short time, have proceeded so far as to be entirely out of the reach of medicine.

Our first object will, of course, be the removal of the cause from which the cachectic state has resulted, supposing that this cause is discovered and is still operating; and we must take care that none other of the causes which have been enumerated be allowed to operate. The patient must be released from all the trammels of study and of business; must breathe a pure atmosphere of a due temperature, and must be cautioned to avoid that habit of stooping which is so common in young persons who labour under this asthenic state, and which is so generally practised by students, who to this posture add the pernicious pressure of the chest against a table or desk. All exertion which entails fatigue must be strictly prohibited; and, if the patient feel weariness in the course of the day, he should recline on a sofa, with the head and chest raised. All inequalities of temperature must be carefully avoided, and the patient should particularly be guarded against sudden alternations of temperature, whether from a cool to a heated, or from a heated to a cool atmosphere. The surface of the body should be protected against the effects of cold; the lower extremities being guarded by warm stockings and thick shoes, while the chest, neck, and arms are covered by a waistcoat of wash-leather. The surface of the chest should be stimulated with the ointment of tartarised antimony, applied repeatedly, so as to produce and to keep up a constant supply of pustules; and, as these begin to die away in one part, they should be called forth in some fresh portion of the surface of the chest, or between the shoulders. This pustular eruption should be kept up so long as the oppressed state of the system continues, or so long as there is any disposition to febrile excitement, to cough, or to uneasiness or oppression of any kind about the respiratory organs. This remedy, in combination with other means, will be found to be powerfully efficacious in removing that indescribable something which fetters and oppresses the general system, while it prevents the commencement of mischief within the chest, and tends, in no slight degree, to arrest and remove any disordered action which may be beginning to develop itself. It is a remedy in common use, but it is too commonly resorted to only when decided mischief has occurred within the chest; whereas, I would wish to enforce the adoption and steady application of it in the simple cachectic state which I have described, and to urge the necessity of keeping up a constant succession of pustules so long as marked indisposition continues.

We now come to the consideration of internal remedies. Our object should be the adoption of those remedies which are calculated to improve the general health, to strengthen the system, to correct any evil tendency in the constitution, to tranquillise the system generally, and to allay local irritation. In the supposed case of the patient now under consideration, these several indications will, in my opinion, be fulfilled by the regular and daily administration of a medicine compounded of Hydrocyanic Acid, Black Drop or Extract of Lettuce, Subcarbonate of Potash or Liquor Potassæ, and Decoction of Myrrh and Lichen. To specify the doses of such familiar remedies may appear to savour of quackery; but I would observe, that, as far as I have seen, full doses of some of these medicines are required to ensure the good effects which they are intended to produce: I may, therefore, be allowed to give the following formula—

℞. Myrrhæ ʒij.; Extracti Glycyrrhizæ ʒiij.; Lichenis ʒvj.; Aquæ Ojss. Decoque ad octarium et cola.

℞. Decocti ut supra ℥jss. ad ℥ij.; Acidi Hydrocyanici gtt. iv.; Guttæ Nigræ gtt. iij.; Potassæ Subcarbonatis gr. viij. M. fiat haustus.

This is my favourite combination, and it affords an admirable remedy in cases such as that now under consideration, when it is employed, as it ought to be, in conjunction with the external application of tartarised antimony. The draught may be given twice or three times a-day.

The patient should drink asses' milk, and should take light, simple nutriment, avoiding stimulants of every kind; never distending the stomach with food, nor fasting too long, nor taking food too frequently. He will, of course, be directed to attend to the state of his bowels, keeping up a natural but gentle action of them by mild unirritating means. Exercise in a carriage, or on horseback, should be enjoined; but the exercise must not be carried so far as to fatigue. The bed of the patient should not be too soft; it should be made so as to present a plane regularly inclined from the head downwards, and it should not be surrounded by closely-drawn curtains.

The foregoing plan of treatment should be strictly enforced, and steadily pursued, so long as the symptoms which have been enumerated present themselves. When all uneasy sensation (whether of pain, of infarction, or of oppression,) is removed from the chest,—when cough is no longer present,—and when all symptoms of febrile excitement have ceased, the stimulating application to the chest may be slowly and cautiously withdrawn; the medicines which have been recommended may be gradually discontinued, and a more generous system of diet may be carefully introduced.

In cases where the symptoms denoting a cachectic state are chiefly those which indicate simple asthenia, the removal of every cause whose operation may induce the cachectic state of which we have been speaking, together with the other cautions which have been laid down, aided by the shower-bath, by friction with salt, by sea-breezes, and by sea-bathing cautiously resorted to, with the further aid which will be derived from the exhibition of four or five grains of Sulphate of Quinine, made into a pill with Extract of Gentian, repeated twice a-day, and of a large tea-spoonful of Liquor Potassæ given three times a-day in table-beer; while, in case of wakefulness or restlessness at night, a pill composed of a grain and a half, or two grains, of Extract of Lettuce, is administered at bedtime: these means will, very generally, be sufficient to re-establish the healthy state of the system. But, if the symptoms of debility maintain their ground in spite of this remedial treatment,—or if there be much restlessness or general irritation present,—or if there be even a suspicion of uneasiness about the chest present,—or if there be present any cough, however trifling it may be,—or if any febrile symptoms present themselves; in either of these cases, recourse should be had to the ointment of tartarised antimony, which should be freely applied, without delay, to the surface of the chest, so as to keep up a constant supply of pustules; and the draught, which has already been recommended, should be substituted for the quinine pill.

It is important that I should remark, that, in cases of this cachectic state of the system, where signs of local disorder are beginning to be superadded to those which spring from the general asthenia; where, for instance, slight cough, obscure uneasiness about the chest, and slight febrile symptoms, present themselves; attempts are too generally made to get rid of the signs of incipient pulmonic disorder by what is termed the strict antiphlogistic treatment, by the employment of which the yielding powers of the system are still further subdued, and the local mischief is too often confirmed. In such cases, abstractions of blood, nauseating doses of Emetic Tartar, or powerful doses of Digitalis, are not unfrequently resorted to as the means of cure ; and they too commonly exhaust the remaining energies of the system, and tend to fix and to hurry onward the local evil against which they are employed. Whereas, in cases of this description, if the practitioner will guard his patient strictly from all sources of mischief; if he, with no sparing hand, will excite pustular eruption on the surface of the chest and between the shoulders, while he administers Prussic Acid and the Black Drop to the extent already specified, in almond emulsion, together with small doses of Nitre combined with the Subcarbonate of Potash; prohibiting stimulating food of every kind; allowing the patient a free supply of asses' milk, of fresh vegetables, and of vegetable puddings; keeping him in a regulated temperature, free from all worry and disturbance; he will, by pursuing this kind of plan, have the satisfaction, in a large proportion of cases, of seeing the signs of pulmonary distress and of febrile excitement gradually disappear; and, by still pursuing this system of treatment for some time after the disappearance of these symptoms, and by then introducing cautiously some mild vegetable tonic, together with a more invigorating plan of diet, he may gradually restore the system to its original healthy condition. In cases such as those which we are now considering, too much anxiety is very commonly shown to get rid of particular symptoms, by means aimed directly at those symptoms; whereas, since symptoms are but the signs, the results, of disease, the only legitimate mode whereby we can get rid of them is by removing the disease which gives rise to them. Acceleration of the pulse, for instance, is very commonly a symptom attendant on the state which we are treating of: it is a symptom dependent on the general irritability of the debilitated system, or on local irritation which has been superadded to the general cachectic condition. Attempts are very generally made, in these cases, to reduce the velocity of the pulse by direct means; but a reduction of the frequency of the pulse is not the removal of disease, it is but the stifling of its warning voice: our object should be the removal of that disease of which the frequency of the pulse is an effect, and we shall find the effect disappear as soon as the cause is withdrawn. As the exhibition of digitalis has been observed, in some cases and under certain circumstances, to be followed by diminished frequency of pulse, that vegetable is usually had recourse to in those cases of incipient pulmonary affection succeeding to general cachexy, which are attended by increased frequency of the pulse. But the free exhibition of digitalis in these cases, if long persevered in, is found, in too many instances, to add to that general debility which was the original founder of the local affection, while it rarely succeeds in subduing that symptomatic acceleration of the circulation, the removal of which, if accomplished by any other means than by a removal of the cause which gave rise to it, cannot answer any good or lasting purpose.

It may happen, indeed, that those first dawnings of pulmonary disorder which so insidiously succeed to general cachexia, may be accompanied by symptoms which indicate slight inflammation of the respiratory organs. In these cases, the immediate imposition of a large blister, with the internal exhibition of Prussic Acid and Nitre, in almond emulsion, with small doses of Ipecacuanha and Extract of Hyoscyamus, may be sufficient to remove those symptoms. Or, in some of these cases, it may seem sufficient to apply leeches to the parietes of the chest, in addition to the other remedies which have been pointed out. But all copious abstractions of blood, and especially general blood-letting, should be avoided in these cases, unless the pressing urgency of inflammatory symptoms imperiously call for them; and, where they are so required, we should not forget to exhibit, after these depletions, the draught of prussic acid and almond emulsion, with the black-drop or lettuce-opium; the prudent and timely administration of which will, by allaying irritation, obviate the necessity of further depletion.

The foregoing remarks have verified the assertion made in the commencement of this paper, that I had no new doctrine to advance, no novel remedy to propose. My object being to call attention to that curable state of cachectic disorder of the system which so commonly lays the foundation of incurable pulmonic disease, it would be foreign to my purpose to enter into the consideration of the more advanced stages of confirmed phthisis.

There is, however, a disordered state of the respiratory organs, which, in its local and general symptoms, is so commonly mistaken for advanced phthisis, but which is, in a great proportion of instances, curable by proper management, that I cannot avoid mentioning it; and I may do so with the greater propriety, because, as this paper has for its object the delineation of curable disorder, and the prevention of incurable pulmonic disease, so may it be allowed to embrace the consideration of a curable malady, which may, in its curable state, be, from its resemblance to phthisis, abandoned as hopeless and irremediable; or, by improper treatment, may be allowed to degenerate into incurable pulmonic disease. The disease to which I allude is bronchial catarrh, in its asthenic or chronic form. In this affection there is troublesome cough, dyspnœa, copious expectoration of thick sputa, which are sometimes streaked with blood: the continued cough, and the unceasing efforts to expectorate, produce and keep up uneasiness of the chest and frequency of the pulse; the appetite becomes much impaired; debility and emaciation ensue, and copious perspiration often occurs; the ankles, in many instances, become œdematous; and there is a strong tendency to effusion within the chest. Affections of this kind, when they have maintained their ground for a considerable length of time, and especially when they occur in habits previously debilitated, may destroy the patient, either by inducing effusion within the chest, or by degenerating into confirmed pulmonic disease. But, in an earlier stage, and not unfrequently even in more advanced stages, these affections may be removed by steady perseverance in the use of remedial means. In these protracted cases of asthenic bronchial affection, there is no remedy more decidedly efficacious than the inhalation of the fumes of tar. Where a more perfect apparatus cannot be procured, every purpose will be answered by placing a small quantity of tar in a small pickling jar, into which is to be introduced a heated piece of iron, having a short wooden handle; inverting over the mouth of the jar a common funnel, and placing the finger of the patient on the orifice of the funnel, by which he will regulate the exit of the fumes. A little practice will teach him the degree of density at which he can respire these fumes, and the degree of heat which should be given to the iron; the temperature of which should be such as to produce dense whitish, and not blackish, fumes. The patient should inhale these fumes, at short intervals, for a few minutes, having recourse to the operation three times a-day. He should, at the same time, apply the ointment of tartarised antimony with freedom to the surface of the chest, so as to procure an ample supply of pustules. He should take also the decoction of myrrh, lichen, and liquorice, with subcarbonate of potash, together with full doses of prussic acid; allaying irritation with the extracts of hyoscyamus and lettuce, or with the black-drop; or he may have recourse to the oleum terebinthinæ, in doses of half a drachm rubbed down with a little syrup of poppies, mixed in decoction of myrrh and cinchona, repeated twice or thrice a-day.

There is another state in which bronchial affection is coupled with great debility, presenting a strong resemblance to confirmed pulmonic disease or to phthisis, but which is, generally speaking, curable. I allude to those cases in which inordinate bronchial secretion, with its common attendants, cough and difficult respiration, occurs in patients who have just emerged from continued fevers of the typhoid type. I have seen not a few cases of this kind, where the harrassing cough, the continual expectoration of thick sputa, the copious perspiration, the wakefulness, the hurried circulation, and the extreme debility of the patient, presented an appearance closely resembling that which accompanies confirmed and incurable pulmonic disease. The remedy on which I have depended in these cases, and on which I have found my reliance well placed, has been opium, given pretty freely and repeatedly: it may be coupled with the prussic acid, and its effects should be supported by mild nutritious diet.

*Old Burlington-street; June* 1826.

